# Heart rate variability and pain sensitivity in people with chronic shoulder pain during passive viewing of shoulder movement pictures

**DOI:** 10.1371/journal.pone.0350902

**Published:** 2026-09-28

**Authors:** Marcela C. Tozzo, Felipe J. J. Reis, Ann Meulders, Hugo C. D. de Souza, Naiara Chinellato, Ana Catarine Veiga, Anamaria S. de Oliveira

**Affiliations:** 1 Department of Health Sciences, Ribeirão Preto Medical School, University of São Paulo, Ribeirão Preto, Brazil; 2 Experimental Health Psychology, Faculty of Psychology and Neuroscience, Maastricht University, Maastricht, The Netherlands; 3 Department of Physiotherapy, Federal Institute of Rio de Janeiro, Rio de Janeiro, Brazil; 4 School of Physical and Occupational Therapy of McGill University, Montreal, Canada; 5 Pain in Motion Research Group, Department of Physiotherapy, Human Physiology and Anatomy, Faculty of Physical Education & Physiotherapy, Vrije Universiteit Brussel, Brussels, Belgium; 6 Health Psychology, KU Leuven, Leuven, Belgium; Ondokuz Mayıs Üniversitesi: Ondokuz Mayis Universitesi, TÜRKIYE

## Abstract

**Background:**

Chronic shoulder pain involves interactions between emotional and autonomic processes that shape pain perception. Movement-related pictures may contribute to autonomic modulation imbalance in musculoskeletal pain. We compared the effects of viewing shoulder movement pictures on autonomic modulation and pressure pain threshold in chronic shoulder pain with pain-free controls.

**Methods:**

This was a cross-sectional study involving 56 chronic shoulder pain and 52 pain-free controls. Participants viewed a sequence of previously validated shoulder movement pictures. Heart rate variability, blood pressure variability, baroreflex sensitivity, pressure pain threshold, and self-reported pain intensity at rest and during the movement (using a numerical pain rating scale) were assessed before, during, and after shoulder movement pictures.

**Results:**

The chronic shoulder pain group showed a reduction in parasympathetic modulation compared to the pain-free control during (high frequency-HF: *p* = 0.04; variance: *p* = 0.00) and after shoulder movement pictures (HF: *p* = 0.03; variance: *p* = 0.01). The time x group interaction was not significant in low frequency (LF) or in the LF/HF ratio and blood pressure variability. The time x group interaction was significant in the baroreflex effectiveness index (*p* < 0.05). The pressure pain threshold was higher in the chronic shoulder pain group after the shoulder movement pictures, with a difference between groups (*p* < 0.001). Numerical pain rating scale did not change in chronic shoulder pain before and after shoulder movement pictures.

**Conclusion:**

Exposure to shoulder movement pictures resulted in reduced parasympathetic modulation and lower pressure pain threshold in chronic shoulder pain.

## 1. Introduction

Chronic Shoulder pain (CSP) is prevalent and arises from a complex interaction of multidimensional factors that modulate pain perception [1–7]. Recently, increased attention has been dedicated to understanding the interaction between pain and emotion [8–11].

Emotions involve responses that arise when an organism detects and reacts to events significant for survival or well-being, encompassing both physiological and behavioral components [12]. In the context of acute pain, negative emotional states and short-term protective behaviors prevent further injury and promote healing. However, when negative emotions persist, they can intensify the perception of threat and promote dysfunctional coping strategies, such as movement avoidance and hypervigilance to pain-related stimuli, perpetuating chronic pain and disability [9,11,13,14]. Effective emotion regulation supports adaptive responses to environmental demands and involves cognitive, behavioral, social, and biological processes that modulate emotions to facilitate goal attainment [15]. Emotion dysregulation has been associated with poorer mental health outcomes and may also amplify pain, sustain its persistence, and contribute to physiological changes, including alterations in autonomic nervous system activity [16–20].

Reciprocal communication between cortico-subcortical networks links cardiac autonomic control with cognitive, emotional, and physiological processes [8,21]. Heart rate variability (HRV), blood pressure variability (BPV), and baroreflex sensitivity (BRS) are widely used indicators to assess autonomic balance and adaptive capacity to environmental and emotional [8,22]. Reduced HRV generally refers to lower values in parasympathetic activity mediated HRV indices and has been associated with several adverse health outcomes. In chronic musculoskeletal pain, lower high-frequency (HF) and root mean square of successive differences (RMSSD), both of which predominantly reflect cardiac parasympathetic activity, have been associated with greater pain intensity, widespread hyperalgesia, central sensitization, and poorer treatment responses [23–25]. Other HRV indices, including low-frequency (LF), the LF/HF ratio, and variance, have also been investigated to characterize autonomic regulation in this population [23,26]. In chronic pain, autonomic impairment may contribute to dysregulated BPV patterns, potentially reflecting altered BRS and increased vascular stiffness [27–29].

Evidence supports that movement-related pictures can evoke emotional and autonomic responses in people with chronic musculoskeletal pain [30–32]. For instance, people with chronic low back pain showed reduced HRV, reduced pressure pain threshold (PPT), and inflated pain self-reports when exposed to movement-related pictures [32]. Those with CSP perceived shoulder movement pictures as negative stimuli [30], and people interpreting them as harmful reported greater shoulder disability [6]. These findings suggest that movement pictures may trigger physiological responses and pain. However, it remains unclear whether passive viewing of shoulder movement pictures can elicit threat-related autonomic responses in CSP. Therefore, the current study aimed to compare: (i) HRV, BPV, and BRS before, during, after a passive viewing task of shoulder movement pictures, and PPT in CSP and pain-free controls; (ii) PPT and self-reported pain intensity before and after the passive viewing task of shoulder movement pictures in both groups. We hypothesized that: (a) CSP would show autonomic alterations (reduced HRV and BRS, increased BPV); (b) these autonomic alterations during, and after picture viewing would be stronger in the CSP than in pain-free controls (i.e., significant group-by-time interactions); (c) CSP would show increased pain sensitivity (lower PPT) and higher self-reported pain intensity after compared to before picture viewing and, (d) CSP would show increases in pain sensitivity (lower PPT) after picture viewing compared with pain-free.

## 2. Methods

### 2.1. Study design, sample size and ethical approval

This cross-sectional study followed the Strengthening the Reporting of Observational Studies in Epidemiology [33,34]. The sample size was calculated based on the comparison of the variables LF and HF, both in their normalized and absolute values, as well as the LF/HF ratio. Among these, the LF/HF ratio required the largest sample size to detect significant differences between groups. The sample size calculation was performed using G*Power software, version 3.1.9.2, and was based on a pilot sample, considering the mean of the LF/HF ratio during the picture viewing period. The pilot study included 10 participants with CSP and 10 pain-free control. The mean and standard deviation (*SD*) of the LF/HF variable were 1.22 (*SD* = 0.56) in the shoulder pain group and 0.84 (*SD* = 0.51) in the control group. Based on these data and using the Student's t-test for comparing means between two independent groups, an effect size of 0.70, a significance level of 5% *(α* = 0.05), and a statistical power of 80% were considered. The calculation indicated the need for 74 participants, with 37 in the CSP group and 37 in the pain-free control group. Additionally, a 12% margin was considered to account for potential sample attrition.

The study was approved by the local Research Ethics Committee and registered (CAAE:67931623.6.0000.5414). All participants provided written informed consent and participated voluntarily in this study.

### 2.2. Participants

Participants were recruited through the public health system of the Municipal Health Department of the city of Ribeirão Preto (São Paulo, Brazil). Additional recruitment occurred through convenience sampling from private clinics and rehabilitation services. The inclusion criteria for participants in the CSP group were: (i) clinical diagnosis of subacromial pain syndrome (ICD-10 M75.1), (ii) shoulder pain persisting for at least three months with an average pain intensity of at least 3/10 in the week prior to assessment, (iii) age over 18 years, and (iv) no physiotherapy treatment in the past month. Pain-free control group was recruited through social media platforms. Participants in the pain-free control group were included based on the following criteria: (i) age over 18 years, (ii) no history of pain or trauma in the shoulder complex or upper limb, and (iii) absence of any other painful condition with active symptoms. The exclusion criteria for both groups were: (i) pregnancy, (ii) diabetes mellitus, (iii) arterial hypertension, (iv) cardiovascular diseases, (v) habitual use of corticosteroids and beta-blocker medications, (vi) history of alcohol abuse in the last 6 months, (vii) Body Mass Index (BMI) above 34.9 kg/m^2^, (viii) neurological diseases, (ix) smoking, and (x) practitioners of high-intensity physical activity (those who practice more than 150 minutes per week of vigorous aerobic activities according to The International Physical Activity Questionnaire (IPAQ) [35,36]. Participants in the CSP and pain-free control were matched for sex and age. All women with an active and regular menstrual cycle were evaluated during the follicular phase, defined as days 6–12 of the menstrual cycle. Participants were instructed to notify the research team on the first day of menstruation, and the experimental assessment was scheduled between days 6 and 12 of the cycle [26,37]. Additionally, because of the psychophysiological measurements, participants were instructed the following 24 hours prior to the assessment: (i) avoid consuming alcohol, caffeine, chocolate, soft drinks, and energy drinks; (ii) refrain from engaging in any form of physical exercise; and (iii) ensure a good night's sleep. These instructions were provided both on the day the assessment was scheduled and 24 hours before the assessment [26,38,39]. A total of 108 participants were included in the study, with 56 participants in the CSP group and 52 participants in the pain-free control group. The flowchart of the study is represented in Fig 1.

**Fig 1:**
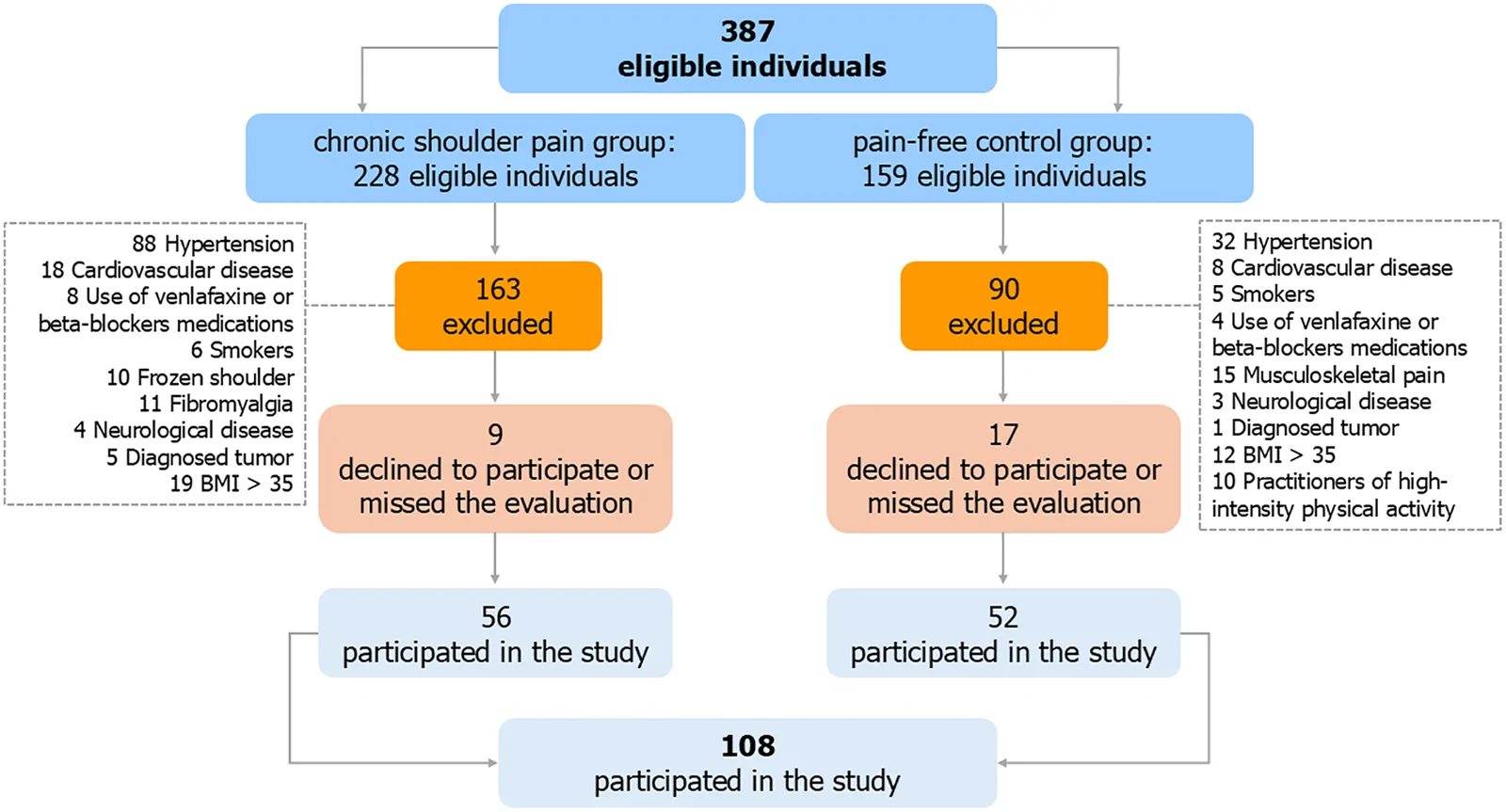
Participant inclusion flowchart for the study.

### 2.3. Stimulus material and apparatus

#### 2.3.1. Shoulder movement pictures.

Twenty-four pictures of daily shoulder-related activities were presented. These pictures were previously evaluated in a motivational impact study in order to compare the hedonic valence and emotional arousal of shoulder movement pictures between people with CSP and pain-free controls [30]. The pictures were rated from negative to neutral for CSP [30]. The description, mean, and SD of valence and arousal of the shoulder movement pictures are also reported in supplementary S1 Table.

#### 2.3.2. Experimental setting and presentation software.

Data was collected between October 2023 and November 2024 at the Department of Health Sciences, Ribeirão Preto Medical School, under standardized conditions, including a temperature of 22°C, artificial lighting, and in the morning between 7:30am and 11:30am. Shoulder movement pictures were presented on a laptop (Dell Inspiron 15 3530) which had a 15-inch screen with a resolution of 1024 x 768 pixels, positioned at the participant’s eye level and direction to ensure comfortable viewing. Microsoft® PowerPoint was used to control the picture presentation.

### 2.4. Procedure

When the participant arrived at the lab, adherence to the instructed recommendations (see Section 2.2, Participants) was checked. Then, the participant received instructions about the experiment and signed the informed consent form. Subsequently, the questionnaires were administered, and the baseline PPT and pain intensity (using the numerical pain rating scale; NPRS) were assessed. After that, the baseline psychophysiological measures were obtained in the seated position. First, the participant was positioned in the supine position on an examination table, and the sensors for HRV, BPV, and BRS recordings were attached. While in this position, the participant received the standardized instructions, which included keeping the left arm still (where the cuff was placed for physiological measurements), minimizing any body movement, remaining awake, and refraining from speaking throughout the assessment [26,38]. Second, the participant was seated in a chair with a backrest, where they remained for a period of 10 minutes. Afterwards, a table was placed in front of the participants to support a notebook [30,32], and the participant received instructions and started the passive viewing task. After viewing the pictures, the participant remained seated for an additional five minutes for the recording of HRV, BPV, and BRS variables [32]. Subsequently, the PPT was reassessed, followed by the administration of NPRS, which was applied exclusively to participants in the CSP group [32,40]. Fig 2.

**Fig 2:**
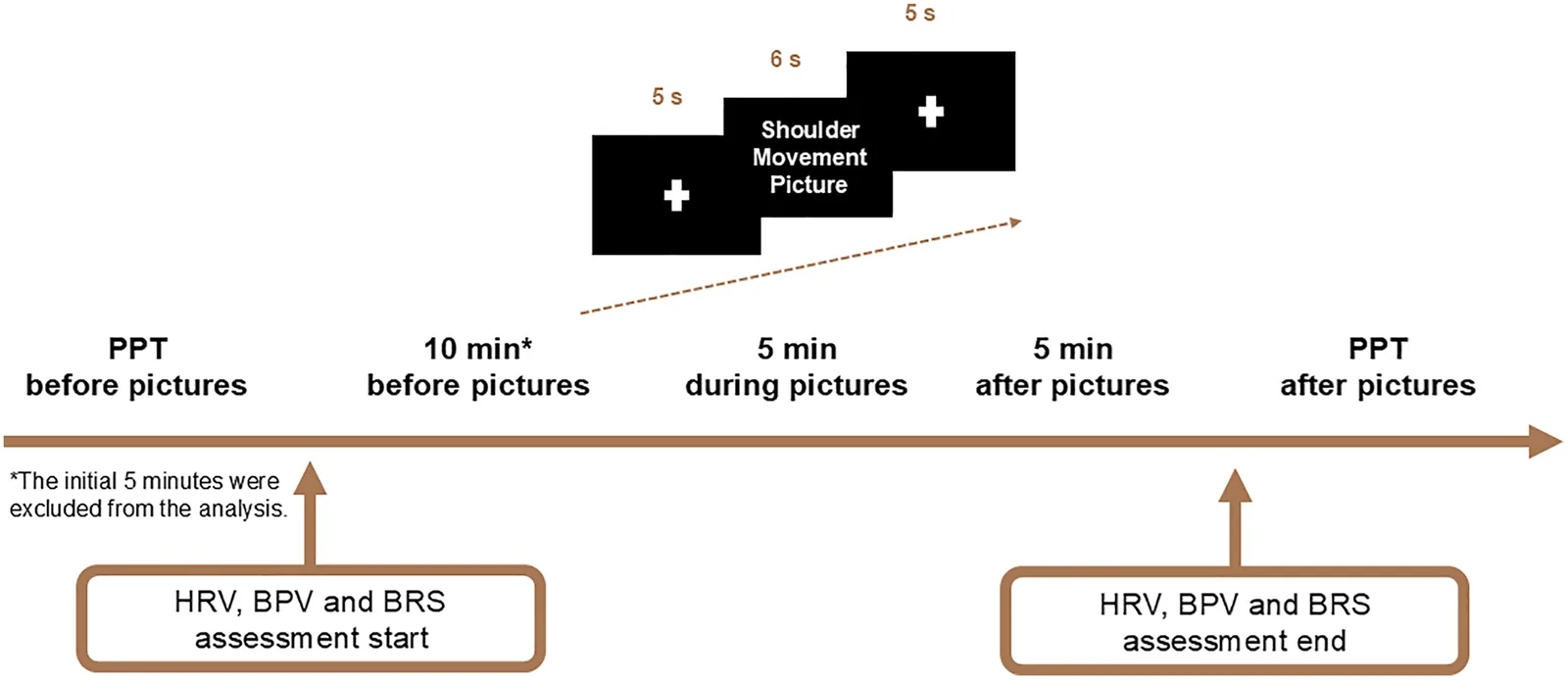
Experimental protocol. Upon arrival, participants completed baseline assessments, including questionnaires, pain intensity, and pressure pain threshold (PPT). The sensors were placed on the participants, and after 10 minutes, psychophysiological recordings (HRV, BPV, and BRS) began. Participants remained seated for 10 minutes; the initial 5 minutes were excluded. Next, participants performed a 5-minute passive visualization task (24 shoulder movement pictures), in which pictures were presented for 6 seconds, interspersed with 5-second fixation crosses. After viewing the shoulder movement pictures, participants remained seated for another 5 minutes for psychophysiological recording. Finally, the PPT and pain intensity (NPRS; only for the group with chronic shoulder pain) were reassessed. The timeline illustrates the sequence and duration of each phase, as well as the physiological measurements collected throughout the procedure.

#### 2.4.1. Passive viewing task.

Participants underwent a 5-minute passive viewing task, after receiving the following instruction: “*Pay close attention to the pictures, as you will be asked questions about them*” [30,32]. The pictures were organized into four presentation blocks (A, B, C, and D). Within each block, the order of the 24 pictures was randomized [30]. Each participant was randomly assigned to view one of these blocks [30]. A fixation point consisting of a white cross (+) on a black background was presented for five seconds at the beginning of the experiment [30,32,41,42]. Next, a picture was displayed full-screen for six seconds [30,32,41,42]. After each picture, a new fixation cross was presented for five seconds (Fig 2).

### 2.5. Outcome measures

#### 2.5.1. Psychophysiological measures.

Regarding the duration of the physiological recordings, participants remained seated for a 10-minute baseline recording. The first 5 minutes were discarded to allow cardiovascular stabilization, and the final 5 minutes were used for baseline HRV, BPV, and BRS analyses. Physiological signals were continuously recorded throughout the 5-minute passive visualization task, and the entire recording was used for analysis. After viewing the shoulder movement pictures, participants remained seated for another 5 minutes for psychophysiological recording and analyzed. Finally, PPT and pain intensity were reassessed (Fig 2).

HRV was obtained from RR intervals (iRR) recorded by electrocardiogram (ECG) (ML866 PowerLab, ADInstruments, Bella Vista, Australia) with modified CM5 shunt configuration, with a sampling frequency of 1000 Hz. This method ensures greater precision and detail of HRV data, especially in spectral and temporal analyses [26,38]. Low frequency (LF; 0.04–0.15 Hz) and high frequency (HF; 0.15–0.4 Hz) bands were calculated, with results expressed in absolute (ms^2^) and normalized (n.u.) units. HRV indices analyzed included LF, high frequency HF, LF/HF ratio, RMSSD and, variance. The LF band reflects a combination of sympathetic and parasympathetic influences; however, higher LF values are typically interpreted as indicating relative sympathovagal imbalance under resting conditions [23,24,26,38,43]. HF band reflects parasympathetic activity [23,24,26,38,43]. The LF/HF ratio has historically been conceptualized as an index of sympathovagal balance, with higher values indicating sympathetic predominance [43]. However, this interpretation has been questioned [23]. A major limitation of the LF/HF ratio is that LF power does not exclusively reflect sympathetic activity but is influenced by multiple factors, including respiration and baroreflex activity [23]. The elevated LF reflects relative sympathovagal imbalance rather than direct sympathetic hyperactivity [23]. The RMSSD reflects the primary time-domain measure used to estimate the parasympathetic mediated changes reflected in HRV. The variance is another measure that reflects HRV and a reduced value of variance represents reduced HRV.

The BPV was obtained from the systolic blood pressure (SBP) values recorded by the Finometer Pro (Finapres Medical System, Amsterdam, the Netherlands). The cuff was positioned on the middle finger of the left upper limb. This device utilizes digital plethysmography and enables continuous blood pressure monitoring and calibration, adjusting the values for the brachial artery using the return-to-flow method and physiological calibration [26,38]. The SBP series were analyzed only in the low frequency band (LF; 0.04–0.15 Hz), with results in absolute values (mmHg^2^). The data collected by Finometer and ECG were transmitted to the computer via the PowerLab4/35 system (ADInstruments, Australia) digitized at a sampling frequency of 1000 Hz, and recorded in LabChart 8.0 software, allowing efficient storage for subsequent analysis. BPV reflects blood pressure fluctuations over time due to the interaction between intrinsic regulation systems and behavioral and environmental factors [44]. An increased BPV shows a vascular reactivity mediated by sympathetic efferent nerves [45]. BPV indices analyzed included variance (mmHg) and, LF absolute (mmHg) and their increased values represents relative sympathovagal imbalance.

BRS was assessed in the time domain using the sequence method which provides three gain indices and one effectiveness index [46,47]. Gain up represents baroreflex sensitivity during increases in blood pressure, whereas gain down reflects sensitivity during decreases in blood pressure. Total gain corresponds to the average gain across all valid sequences, indicating overall baroreflex sensitivity. The baroreflex effectiveness index (BEI) expresses the proportion of blood pressure ramps that elicit a reflex change in pulse interval, reflecting baroreflex effectiveness [46,47]. Reduced values of gain up, gain down, and total gain indicate lower baroreflex sensitivity, whereas reduced BEI values indicate lower baroreflex effectiveness. Taken together, reductions in these indices suggest decreased parasympathetic modulation, a relative increase in sympathetic activity, and diminished baroreflex sensitivity and effectiveness [46,47]. These alterations are characterized by a reduced capacity of the cardiovascular system to modulate heart rate in response to spontaneous fluctuations in blood pressure [46,47].

HRV, BPV and BRS analyses were performed using CardioSeries v2.4 software (http://sites.google.com/site/cardioseries), developed by the University of São Paulo [48]. Before spectral and baroreflex analyses, both the beat-to-beat iRR and SBP series were resampled using cubic spline interpolation at 3 Hz to obtain evenly spaced time series suitable for frequency-domain analysis. The interpolated RR and SBP series were then divided into semi-overlapping sets of 512 data points with 50% overlap (Welch protocol) [26,38]. Before spectral analysis, stationary segments were visually inspected, and segments containing artifacts or transient disturbances were excluded. Each stationary segment was then subjected to spectral analysis using Fast Fourier Transform after application of a Hanning window. The iRR time series were integrated into the low-frequency (LF; 0.04–0.15 Hz) and high-frequency (HF; 0.15–0.40 Hz) bands, with results expressed in absolute (ms^2^) and normalized units (n.u.), whereas the SBP time series were analyzed only in the LF band (0.04–0.15 Hz), with results expressed in absolute values (mmHg^2^) [26,38].

#### 2.5.2. Pressure Pain Threshold.

The PPT was measured using a KRATOS algometer – model DDK. The 1 cm^2^ tip was applied perpendicularly to the skin, and pressure was gradually increased until the participant indicated to first feel pain. Participants were instructed to say “stop” as soon as the pressure sensation became painful. They were specifically instructed to indicate the onset of pain rather than the maximum level of pain they could tolerate [32,40]. PPT was assessed at four sites: the right and left middle deltoid, and the right and left anterior tibialis muscles [49,50]. To standardize the procedure, measurements were collected by alternating sides: the first three measures were obtained at the right deltoid and left tibialis (alternated across trials), followed by three measures at the left deltoid and right tibialis (also alternated). A 30-second interval was applied between attempts, and the mean of the three measures for each site was used for analysis [51]. Lower PPT values indicate greater pain sensitivity.

#### 2.5.3. Pain intensity.

The NPRS was used to assess pain intensity [52,53]. The NPRS ranges from 0 to 10, where 0 represents “no pain” and 10 represents “worst possible pain” [52–54]. Pain intensity was assessed at rest using the question: *“What is the intensity of the shoulder pain you are feeling right now, with your arm still, at rest, alongside your body?*”. Pain intensity during movement was assessed while participants actively performed shoulder abduction in the frontal plane through their full available range of motion. Participants were instructed to report the maximum intensity of shoulder pain experienced while performing the movement using the NPRS. Participants were asked: “*What was the maximum intensity of shoulder pain you experienced while elevating your arm?*” Participants were not required to maintain the arm at a specific angle or perform an isometric contraction. Pain intensity referred to the maximum pain experienced throughout the movement.

Also, the pain intensity within the past week was assessed at baseline as part of the initial evaluation. Pain intensity was assessed only in the CSP group, as participants in the pain-free group met the inclusion criteria of having no history of pain or trauma in the shoulder complex or upper limb and no other painful condition with active symptoms.

### 2.6. Questionnaires

An initial interview was conducted to collect sociodemographic data and characterize the sample. Information about previous medical diagnoses of anxiety or depression was also obtained. This interview took place at the beginning of the experimental session, after signing the informed consent form and before starting the experimental procedures. After completing this initial assessment, the following instruments were administered to the CSP group:

(1) The Brazilian version of the Shoulder Pain and Disability Index (SPADI), which assesses disability levels related to shoulder pain [55]. It consists of 13 items distributed across pain and function domains, scored on a numerical rating scale from 0 to 10 points. A higher score indicates worse shoulder disability levels [55]. The measurement properties of the questionnaire have been tested, demonstrating that it is a reliable and valid tool for assessing shoulder-related disability [55];
(2) The Avoidance Daily Activity Photo (ADAP) Shoulder Scale assesses the avoidance behavior of daily movements in people with shoulder pain. The scale consists of 15 pictures representing people performing daily activities with the upper limb, distributed in 3 domains: free movement, high effort, and self-care [56,57]. Participants are instructed to answer the following question for each pictures shown: “*Considering your shoulder pain, how much would you avoid the activity shown in the picture if you had to do it now?*” Each picture is presented with a numerical scale from 0 to 10, with 0 representing “no avoidance” and 10 “extreme avoidance”. The total score on the scale ranges from 0 to 100, with higher values indicating the presence of avoidance behavior [56,57]. The scale has demonstrated high reliability, strong internal consistency, and validity as a measure of avoidance behavior in people with shoulder pain [56,57];
(3) The Brazilian Portuguese Pain Catastrophizing Scale (BP-PCS) is a self-administered questionnaire consisting of 13 items for assessing pain-related worrying [58]. The BP-PCS is divided into the following 3 domains: magnification, rumination, and helplessness. The questionnaire is filled out based on the participant’s thoughts and feelings about pain [58].

In addition, participants in both groups completed the Patient Health Questionnaire-9 (PHQ-9). The PHQ-9 screens for symptoms of depression consists of nine items representing symptoms of depression and is scored on a four-point scale (0 = not at all and 3 = almost every day) [59,60]. The PHQ-9 has been validated and demonstrated high reliability [59,60].

### 2.7. Statistical analysis

Statistical analyses were performed using R software (version 2025.05.0 + 496). Categorical variables were described as frequencies and percentages, while continuous variables were presented as mean and standard deviation or median and interquartile range, as appropriate. Data distributions were examined through the use of histograms and the Shapiro–Wilk test [61]. To assess homogeneity, the Levene test was used. The homoscedasticity was inspected using scatter plots and the model residuals were formally assessed using Levene's test for homogeneity of variance (center = median), applied to simulated residuals obtained via the DHARMa package [61,62], comparing residual variance between groups and across experimental phases. Levene's test was not significant for any model (all p > 0.05), indicating no evidence of heteroscedasticity.

For the between-group analyses we used generalized linear mixed models (GLMMs) implemented with the glmmTMB package. This approach was selected due to the non-normal distribution of the dependent variables, verified through visual inspection of histograms and Cullen and Frey plots. A GLMM with a Gamma distribution and log link function was fitted [63]. For models with a statistically significant group-by-time interaction, effect magnitude was expressed as rate ratios (RR) with 95% confidence intervals, obtained from exponentiated model coefficients, which represent the appropriate measure of effect for Gamma-distributed outcomes on the log scale. The assumption of the absence of multicollinearity was assessed by calculating the Variance Inflation Factor (VIF) and was considered satisfied when all VIF values were below 5 [64].

The model included the following fixed effects: group (CSP vs. pain-free control), time (before, during and after pictures), and the group vs. time interaction. Random intercepts by subject were incorporated to account for the repeated measures structure. The significance of fixed effects was assessed through deviance analysis using Type III Wald chi-square tests [63]. When a statistically significant group vs. time interaction was identified (*p* < 0.05), post hoc analyses were conducted by calculating estimated marginal means (EMMeans) using the emmeans package. Pairwise comparisons were performed: between groups at each time point, and between time points within each group, with *p*-value adjustment using Tukey’s method for multiple comparisons. The significance level was set at 5% (α = 0.05).

A 2x2 mixed ANOVA was conducted to compare mean pressure pain thresholds before and after picture viewing (within-subject independent variable) across different groups (between-subject independent variable). Statistically significant main effects or interactions were followed by pairwise comparisons with Bonferroni correction.

The non-parametric Wilcoxon signed-rank test was conducted to assess differences in pain intensity before and after picture viewing in the CSP group only. Given that the p-value is influenced by sample size, the effect size r, appropriate for the Wilcoxon test, is also reported [65]. *R values can be interpreted as small (r ≥ 0.1), medium (r ≥ 0.3), or large (r ≥ 0.5)* [66]. Exploratory Spearman correlation analyses were performed to investigate the associations between changes in autonomic modulation, PPT and pain intensity during picture exposure. Change scores (Δ) were calculated as during pictures minus before pictures HRV, and BEI, and after pictures minus before pictures for PPT and pain intensity. The Spearman correlation coefficient (ρ) is an effect size measure whose absolute value can be interpreted as weak (ρ ≥ 0.30), moderate (ρ ≥ 0.50), high (ρ ≥ 0.70), or very high (ρ ≥ 0.90) [67]. The complete correlation matrix is presented in Supporting Information S1 Fig.

## 3. Results

### 3.1. Participants

Sociodemographic and clinical characteristics of participants are presented in Table 1.

**Table 1:** Sociodemographic and clinical characteristics of participants (*N* = 108).

	Chronic Shoulder Pain Group (n = 56)	Pain-free Control Group (n = 52)
**Age (years), mean (SD)**	44.6 (10.6)	40.3 (12.0)
**Sex, n (%)** *Female* *Male*	39 (69.6%) 17 (30.4%)	37 (71.1%) 15 (28.8%)
**Weight (kg), mean (SD)**	71.1 (12.7)	70.7 (13.8)
**Height (cm), mean (SD)**	162 (9.1)*	165 (7.8)*
**BMI, mean (SD)**	26.7 (3.8)	25.3 (3.8)
**Dominance, n (%)** *Right* *Left*	52 (92.8%) 4 (7.2%)	49 (94.3%) 3 (5.7%)
**PHQ-9, mean (SD)**	4.7 (5.6)*	1.0 (2.9)*
**Painful shoulder, n (%)** *Dominant* *Non-dominant*	35 (62.5%) 21 (37.5%)	
**Symptom Duration (months), mean (SD)**	28.2 (28.9)	
**NPRS, mean (SD)** *Present pain at rest* *Pain during movement* *Pain within the past week*	2.2 (2.9) 5.7 (2.5) 8.4 (1.9)	
**SPADI, mean (SD)** *Total* *Function* *Pain*	61.2 (22.6) 56.4 (24.1) 68.8 (23.8)	
**ADAP Shoulder Scale, mean (SD)** *Total* *Free movement* *High effort* *Self-care*	48.9 (25.2) 45.6 (32.5) 65.3 (27.7) 16.0 (27.6)	
**PCS, mean (SD)** *Total* *Magnification* *Rumination* *Helplessness*	21.9 (13.1) 4.9 (3.8) 8.2 (4.2) 8.7 (5.8)	

Note – SD: standard deviation; n: number of participants; kg: kilograms; cm: centimeters; BMI: body mass index; PHQ-9: Patient Health Questionnaire-9; NPRS: Numerical Pain Rating Scale; SPADI: Shoulder Pain And Disability Index; ADAP: Avoidance Daily Activity Photo Shoulder Scale; PCS: Pain Catastrophizing Scale; *p < 0.05 between groups.

### 3.2. Heart rate variability

The median and interquartile range of all variables are presented in Table 2.

**Table 2:** Descriptive statistics of the variables by group and experimental time (*N* = 108).

Chronic Shoulder Pain Group (*n* = 56)				Pain-free Control Group (*n* = 52)		
Median (interquartile range)				Median (interquartile range)		
	Before pictures	During pictures	After pictures	Before pictures	During pictures	After pictures
**Frequency domain**						
LF (ms2)	387(156–791)	348(139–872)	432(206–895)	620(335–1453)	523(228–1411)	779(307–1404)
HF (ms2)	242(130–452)	241(127–578)	255(122–542)	529(243–905)	561(209–878)	476(242–777)
LF nu (%)	56(45–69)	53.5(40.8 to 68.5)	59.3(43.8 to 73.2)	56.5(46.8 to 65.2)	54.5 (41.8 to 67.2)	64.5(49 to 72.8)
HF nu (%)	44(31–55)	46.5(32.8 to 59.2)	40.6(26.8 to 56.2)	43.5(34.8 to 53.2)	48 (32.8 to 59.5)	35.5(27.2 to 51)
LF/HF	1.38(0.92 to 2.66)	1.26(0.728 to 2.25)	1.66(0.86 to 3.02)	1.6(0.9 to 2.26)	1.26 (0.73 to 2.3)	2.08 (1.06 to 2.9)
Variance (ms²)	1867(939–3637)	1402(660–2720)	2098(1062–3850)	2682(1512–4520)	2696 (1202–4532)	3121(1804–4921)
**Time domain**						
RMSSD (ms)	28.4(22.4 to 40.2)	28.4(21.2 to 44)	30.3(22.3 to 40.3)	36.8(27.2 to 47.6)	37.0(25.5 to 45.3)	36.2(25.5 to 49.0)
**Blood pressure variability**						
Variance(mmHg²)	32.8(23.8 to 46.7)	24.9(15.3 to 32.8)	35.4(24.0 to 46.2)	35.4(23.2 to 47.8)	28.4(19.5 to 36.2)	44.4(26.8 to 53.8)
LF abs (mmHg²)	9.66(5.35 to 11.5)	8.20(5.38 to 10.3)	10.5(5.54 to 13.4)	11.4(6.42 to 13.8)	7.57(5.31 to 12.4)	11.7(7.82 to 13.5)
**Baroreflex sensitivity**						
BEI	0.469(0.36 to 0.572)	0.506(0.401 to 0.608)	0.497(0.401 to 0.59)	0.628(0.476 to 0.757)	0.627(0.492 to 0.754)	0.56(0.47 to 0.701)
Gain up (ms/mmHg)	8.7(5.69 to 11.3)	9.79(5.89 to 12.0)	9.90(5.66 to 11.2)	11.7(7.07 to 15.9)	11.1(7.27 to 13.1)	11.4(6.34 to 13.2)
Gain down (ms/mmHg)	9.93(6.57 to 11.3)	9.43(6.66 to 11.8)	10.2(7.17 to 11.4)	12.6(7.48 to 14.6)	12.6(8.29 to 15.1)	12.5(8.25 to 15.1)
Total gain (ms/mmHg)	9.40(6.36 to 11.2)	9.44(6.38 to 11.8)	10.0(6.72 to 10.8)	12.0(7.84 to 15.8)	11.9(7.68 to 13.6)	11.7(7.18 to 13.5)

Note – N, number of participants; HF, high frequency; LF, low frequency; BEI, baroreflex effectiveness index; ms2, milliseconds; nu, normalized units; SD, standard deviation; RMSSD, root mean square of successive differences; ms/mmHg, milliseconds/millimeter of mercury; up, baroreflex sequence with progressive increases in blood pressure followed by progressive increases in pulse interval; down, baroreflex sequence with progressive decreases in blood pressure followed by progressive decreases in pulse interval.

The HF abs variable (ms^2^) showed significant main effects for group, χ²(1) = 6.46, *p* = .011, and time, χ²(3) = 30.88, *p* < 0.001, as well as a significant group-by-time interaction, χ²(3) = 14.09, *p* = 0.002, suggesting that variation throughout the experiment differed between groups. Marginal pairwise comparisons showing lower HF abs values in the CSP group than in the pain-free group before, during, and after picture viewing are presented in Table 3.

**Table 3:** Between-group pairwise comparisons across experimental phases for variables showing significant group-by-time interactions (N = 108).

Variables	Rate ratio (95% CI)	Z ratio	P value
HF (ms2)			
Before pictures	**0.571(0.446 to 0.732)**	**−2.542**	0.011*
During pictures	**0.642(0.486 to 0.848)**	**−2.010**	0.0445*
After pictures	**0.632(0.481 to 0.83)**	**−2.080**	0.0376*
Variance (ms²)			
Before pictures	0.742(0.587 to 0.939)	−1.798	0.072
During pictures	**0.635(0.486 to 0.829)**	**−2.732**	0.0063*
After pictures	**0.668(0.513 to 0.869)**	**−2.426**	0.0153*
BEI			
Before pictures	**0.782(0.669 to 0.914)**	**−3.256**	0.0011*
During pictures	**0.860(0.744 to 0.994)**	**−1.999**	0.045*
After pictures	0.914(0.799 to 1.045)	−1.195	0.2323
Total gain (ms/mmHg)			
Before pictures	0.824(0.70 to 1.01)	−1.873	0.061
During pictures	0.876(0.73 to 1.05)	−1.285	0.199
After pictures	0.936(0.77 to 1.13)	−0.637	0.524

Note – HF, High Frequency component of heart rate variability; BEI, Baroreflex Effectiveness Index; CI, Confidence Interval; ms^2^, milliseconds squared; mmHg, millimeters of mercury. Bold values indicate statistically significant pairwise comparisons. *P < 0.05.

The variance variable (ms^2^) showed a significant main effect of time, χ^2^(3) = 66.08, *p* = 0.000, and a significant group-by-time interaction, χ^2^(3) = 10.84, *p* = 0.013. The main effect of group was not significant, χ^2^(1) = 3.23, *p* = 0.072. Marginal pairwise comparisons showing lower variance (ms^2^) values in the CSP group than in the pain-free group before, during, and after picture viewing are presented in Table 3.

The deviance analysis of the LF abs variable (ms^2^) (Type III Wald χ^2^ tests) revealed significant main effects for group, χ^2^(1) = 7.77, *p* = 0.005, and time, χ^2^(3) = 44.88, *p* < 0.001. The group-by-time interaction did not reach statistical significance, χ^2^(3) = 5.94, *p* = 0.114. The other variables (LF nu, HF nu, LF/HF, and RMSSD) did not show significant main effects of group or group-by-time interactions (*p* > 0.05). The significant group-by-time interaction indicates that the trajectory of variables across experimental phases, including the transition from baseline to picture exposure and from exposure to recovery, differed significantly between groups; Tables 3 and 4 present the corresponding between- and within-group contrasts underlying this interaction.

**Table 4:** Within-group pairwise comparisons across experimental phases for variables showing significant group-by-time interactions (N = 108).

Group	Before Pictures vs. During Pictures Ratio (95% CI)	*p*	Before Pictures vs. After Pictures Ratio (95% CI)	*p*	During Pictures vs. After Pictures Ratio (95% CI)	*p*
HF (ms2)						
CSP	0.99 (0.74–1.32)	0.996	**1.51 (1.13–2.00)**	0.002*	**1.52 (1.14–2.03)**	0.002*
Pain-free Control	1.15 (0.86–1.54)	0.506	1.03 (0.77–1.38)	0.969	0.90 (0.67–1.19)	0.646
Variance (ms²)						
CSP	0.92 (0.72–1.17)	0.677	1.15 (0.91–1.46)	0.359	1.25 (0.99–1.58)	0.063
Pain-free Control	1.25 (0.98–1.59)	0.090	1.10 (0.86–1.40)	0.641	0.88 (0.69–1.12)	0.446
BEI						
CSP	1.06 (0.93–1.21)	0.544	**1.15 (1.01–1.31)**	0.033*	1.08 (0.95–1.23)	0.317
Pain-free Control	0.96 (0.84–1.10)	0.744	0.99 (0.87–1.13)	0.981	1.03 (0.90–1.18)	0.848
Total gain (ms/mmHg)						
CSP	0.99 (0.94–1.05)	0.949	1.04 (0.98–1.09)	0.268	1.04 (0.99–1.10)	0.152
Pain-free Control	1.02 (0.97–1.08)	0.583	1.02 (0.97–1.08)	0.653	1.00 (0.94–1.05)	0.993

Note – HF, High Frequency component of heart rate variability; BEI, Baroreflex Effectiveness Index; CI, Confidence Interval; ms^2^, milliseconds squared; mmHg, millimeters of mercury; CSP, chronic shoulder pain group. Bold values indicate statistically significant pairwise comparisons. *P < 0.05.

### 3.3. Blood pressure variability

The median and interquartile range of all variables are presented in Table 2. The deviance analysis (Type III Wald χ^2^ tests) for variance indicated a significant effect of time, χ^2^(3) = 47.84, *p* < 0.001, as well as for the LF abs variable, χ^2^(3) = 21.92, *p* < 0.001. However, these variables did not show significant main effects for group or group-by-time interactions (*p* > 0.05).

### 3.4. Baroreflex sensitivity

The median and interquartile range of all variables are presented in Table 2. The BEI variable showed significant main effects for group, χ^2^(1) = 10.60, *p* = 0.001, and time, χ^2^(3) = 9.26, *p* = 0.026, as well as a significant group-by-time interaction, χ^2^(3) = 10.24, *p* = 0.017, indicating that the BEI differed between groups. Comparisons revealed that before picture viewing, the CSP group presented significantly lower BEI. Marginal pairwise comparisons showing lower BEI values in the CSP group than in the pain-free group before, during, and after picture viewing are presented in Table 3.

The total gain variable indicated a marginally significant effect for group, χ^2^(1) = 3.51, *p* = 0.061, and time, χ^2^(3) = 6.63, *p* = 0.085, as well as a significant group-by-time interaction, χ^2^(3) = 8.25, *p* = 0.041, suggesting that the variation of BRS throughout the experiment differed between groups. Marginal pairwise comparisons between groups for total gain before, during, and after picture viewing are presented in Table 3. However, these comparisons did not reveal statistically significant differences *(p* > 0.05) (Table 3).

The other variables (gain up and gain down) did not show significant main effects for group or group-by-time interactions (*p* > 0.05). Table 4 reports within-group pairwise comparisons across experimental phases for variables showing significant group-by-time interactions.

### 3.5. Pressure pain threshold

Repeated-measures ANOVA revealed significant differences in the dominant deltoid for group F(1,212) = 41.5, p < 0.001, and time F(1,212) = 4.37, p = 0.03, but not for group × time interaction F(1,212) = 1.68, p = 0.19. For the non-dominant deltoid, significant effects were also observed for group F(1,212) = 8.00, p < 0.001, and time F(1,212) = 7.59, p < 0.001, but not for the group × time interaction F(1,212) = 2.54, p = 0.11. Regarding the dominant tibialis anterior, there were significant effects of group F(1,212) = 32.74, p < 0.001, but not for time F(1,212) = 1.53, p = 0.21, neither for the group × time interaction F(1,212) = 3.65, p = 0.05. Finally, the non-dominant tibialis anterior also showed significant effects of group F(1,212) = 27.30, p < 0.001, but not for time F(1,212) = 2.73, p = 0.09, or the group × time interaction F(1,212) = 3.46, p = 0.06. Bonferroni post-hoc comparisons indicated that the CSP group exhibited lower pressure pain thresholds at all sites after picture viewing compared to the pain-free control group. Detailed results are presented in Table 5 and Fig 3 illustrates the changes in pressure pain threshold before and after picture viewing for both groups.

**Table 5:** Unadjusted Mean (SD) and Adjusted Mean Difference and 95% CIs for the PPT Before and After Pictures Visualization. Chronic Shoulder Pain Group (*N* = 56) and Pain-free Control Group (*N* = 52).

Region	Group	Before Pictures Mean (SD)	After Pictures Mean (SD)	Within-Group p-value	Adjusted Between-Group Difference After Pictures (95% CI)	Between-Group After Pictures p-value
**Deltoid – Dominant**						
	CSP	3.3 (1.4)	2.4 (1.4)	0.010*	**–1.89 (–2. 7 to –1.20)**	<0.0001*
	Pain-free Control	4.5 (2.0)	4.3 (2.2)	0.58		
**Deltoid – Non-dominant**						
	CSP	3.4 (1.9)	2.4 (1.4)	0.006*	**−1.82 (−2.52 to −1.11)**	<0.0001*
	Pain-free Control	4.4 (1.9)	4.3 (2.0)	0.659		
**Tibialis Anterior – Dominant**						
	CSP	5.4 (2.2)	4.4 (2.1)	0.023*	**−2.27 (−3.10 to −1.44)**	<0.0001*
	Pain-free Control	6.5 (2.2)	6.7 (2.1)	0.642		
**Tibialis Anterior – Non-dominant**						
	CSP	5.5 (2.1)	4.4 (2.1)	0.011*	**−2.18 (−3.04 to −1.32)**	<0.0001*
	Pain-free Control	6.5 (2.3)	6.6 (2.4)	0.885		

Note – CI, confidence interval; PPT, pressure pain threshold; SD, standard deviation; N, number of participants; CSP, Chronic Shoulder Pain. Bold values indicate statistically significant pairwise comparisons. *P < 0.05.

**Fig 3:**
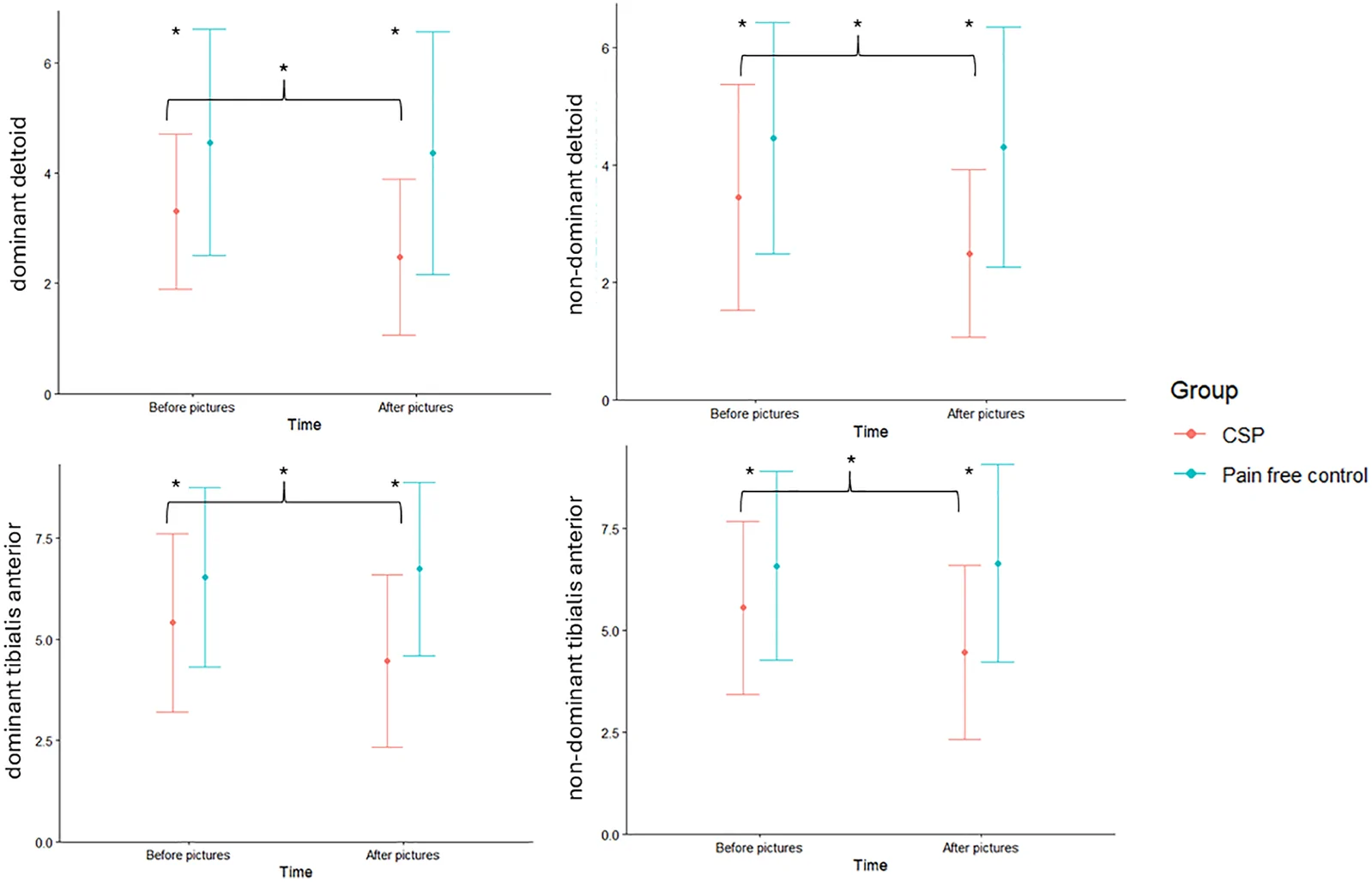
Pressure pain threshold (PPT) before and after picture viewing in participants with chronic shoulder pain (CSP) and pain-free controls. Data are presented as mean ± standard deviation for the dominant and non-dominant deltoid muscles and the dominant and non-dominant tibialis anterior muscles. *p < .05.

### 3.6. Pain intensity

Pain intensity was assessed at rest and during movement before and after the picture-viewing task using the NPRS in the CSP group (n = 56). No significant differences were observed when comparing baseline with after-picture viewing either at rest, *p* = 0.802, or during movement, *p* = 0.662. Detailed results are presented in Table 6.

**Table 6:** Numerical Pain Rating Scale before and after viewing pictures in chronic shoulder pain group (N = 56).

	Chronic Shoulder Pain Group (n = 56)Median (interquartile range)			
	Before Pictures	After pictures	P-value	Effect size r (CI95%)
At rest	0 (0 to 4.25)	0 (0–4)	0.802	−0.04 (−0.45 to 0.32)
At movement	6 (3–8)	6 (3–8)	0.662	−0.08 (−0.42 to 0.27)

Note – CI, confidence interval.

### 3.7. Exploratory correlations between autonomic modulation, PPT and pain intensity

The Spearman correlation analysis indicated that no meaningful associations were observed between changes in autonomic modulation variables (HRV indices and BEI) and changes in PPT or pain intensity during picture viewing minus before picture viewing (all p > 0.05). The complete correlation matrix is presented in Supplementary S1 Fig.

## 4. Discussion

### 4.1. Main findings

The main aims of this study were to compare HRV, BPV, and BRS before, during, after a passive viewing task of shoulder movement pictures, and PPT in people with CSP and pain-free controls. This study also examined changes in PPT before and after the passive viewing of shoulder movement pictures in both groups, as well as changes in self-reported pain intensity before and after the task in the CSP group only. Our hypotheses were partially confirmed. We hypothesized that individuals with CSP would show autonomic alterations (reduced HRV and BRS, increased BPV) and increased pain sensitivity during and after picture viewing. We also expected these effects to be greater than those observed in pain-free controls. Consistent with these hypotheses, individuals with CSP showed reduced HRV, evidenced by lower HF and HRV variance, and reduced BRS, evidenced by a lower baroreflex effectiveness index (BEI). These differences were evident during and after picture viewing compared with pain-free controls. However, we did not observe differences between groups in LF or BPV indices (LF absolute power and BPV variance). Consistent with our pain-related hypotheses, the CSP group exhibited significantly lower PPT across all assessed regions, with a further reduction after exposure to shoulder movement pictures. These results may suggest increased nociceptive sensitization induced by picture viewing. This pattern was not observed in pain-free controls, whose PPT values remained stable throughout the protocol. In contrast to our expectations, self-reported pain intensity did not differ in the CSP group before and after picture viewing.

Overall, our findings partially align with those of a study that assessed HRV in people with chronic low back pain during the passive viewing of movement pictures [32]. Our findings indicate that CSP exhibit reduced HRV during exposure to shoulder movement pictures. This effect is evidenced by a significant decrease in absolute HF values during and after the shoulder movement pictures, whereas in the study involving people with chronic low back pain, the authors observed a similar pattern based on HF values. Both studies converge on the notion that chronic pain is associated by a reduction in parasympathetic activity [32]. Our study found a reduction in variance in the CSP group during and after viewing shoulder movement pictures. Variance is another index of HRV and its reduction reflects diminished autonomic modulation in CSP when exposed to negative visual stimuli [8,43]. However, we did not find significant differences in LF power or the LF/HF ratio, suggesting that the observed autonomic alterations were primarily reflected in parasympathetic activity mediated HRV indices [26] In one study, people with chronic neck pain did not show differences in HRV variables compared to pain-free controls during acute experimental pain induced by pressure and cold stimulation [40]. This suggests that not all musculoskeletal pain conditions exhibit relative sympathovagal imbalance to negative stimuli. Thus, our findings point to a parasympathetic activity hypoactivity. Reduced parasympathetic activity may contribute to impaired anti-inflammatory regulation through reduced cholinergic anti-inflammatory activity, potentially favoring the maintenance of a pro-inflammatory state [68] and may also be associated with increased sensitivity to painful stimuli [69–71]. Such autonomic alterations have also been suggested as potential contributing factors to the maintenance of shoulder disorders and the development of frozen shoulder [70]. Evidence from chronic low back pain suggests that reduced parasympathetic activity may be associated with greater pain-related fear and pain-related avoidance behavior [71,72]. Although these mechanisms cannot be confirmed by the present study, they may help explain the relationship between autonomic imbalance and CSP.

Unlike chronic low back pain, which is often associated with greater impact on daily activities, CSP may not evoke the same level of perceived threat or emotional burden during the passive viewing of movement pictures. Moreover, participants were not explicitly instructed to imagine themselves performing the motor tasks, which might have decreased emotional engagement [73].

In the present study, no significant differences in BPV were observed between the groups. Unlike HRV, which is influenced by both sympathetic and parasympathetic components, BPV appears to be primarily regulated by sympathetic activity [74]. As a result, even in the presence of a negative visual stimulus for people with CSP and a reduction in parasympathetic activity, BPV may remain unchanged due to its greater reliance on sympathetic control [74]. These findings suggest that BPV may have limited sensitivity to capture autonomic alterations elicited by shoulder movement pictures in CSP, compared with HRV [72,75]. Thus, BPV may not be the most appropriate measure for assessing autonomic alterations elicited by shoulder movement pictures in CSP, reinforcing the importance of using markers that are more sensitive to parasympathetic modulation [72,75].

Regarding BRS, we did not find a group-by-time interaction in the gain variables (up, down and total). However, similar to BPV, it may not be an appropriate physiological measure for assessing autonomic alterations elicited by negative visual stimulus in CSP.

Regarding PPT, our findings align with those from the chronic low back pain study [32]. In our study, the CSP group showed a significant reduction in PPT across all assessed regions after the passive viewing task, while the pain-free control group maintained stable values. These findings support the hypothesis that the viewing of shoulder movement pictures may act as an emotional stressor capable of amplifying pain sensitivity, possibly through mechanisms of central facilitation [76–79]. Regarding pain intensity, unlike the findings of Bandeira et al., who reported a significant increase in pain intensity after the viewing task, pain intensity did not change significantly in our CSP sample [32]. These results suggest that although the viewing of shoulder movement pictures can modulate pain sensitivity in people with CSP, it may not be sufficient to immediately alter self-reported pain.

### 4.2. Limitations

This study has some limitations that should be acknowledged. Although participants were not instructed to imagine performing the movements, this could not be fully controlled. It is possible that explicit instructions to engage in motor imagery could have increased the emotional salience of the stimuli [73]. Respiratory activity was not monitored during the experimental protocol. Therefore, its potential influence on HRV, BPV, and BRS measurements could not be controlled, which should be considered when interpreting the findings. The generalization of our results should be made with caution, as the exclusion criteria adopted, although necessary to control confounding variables, led to the exclusion of a considerable number of participants. The exclusion criteria may have limited the representativeness of the sample in relation to the broader clinical population with CSP. Moreover, self-reported fear of movement-related pain or perceived threat during the task were not assessed, which could have helped interpret the absence of changes in some autonomic variables. Another limitation concerns the study design, as neutral and negative shoulder movement pictures were presented in a intermixed rather than in separate blocks. Because the task was not designed to isolate emotional effects, it was not possible to determine whether negative stimuli would have elicited stronger autonomic responses than neutral ones. Importantly, presenting both picture categories together may have diluted emotional contrasts and reduced the likelihood of detecting significant autonomic differences.

### 4.3. Clinical applications and future research

The findings of this study support the importance of considering the influence of shoulder movement on autonomic and sensory mechanisms in people with CSP. Shoulder movement pictures may function as symbolically relevant cues associated with anticipated pain or harm, thereby eliciting autonomic responses (reduction in parasympathetic modulation and the increase in pain sensitivity) characteristic of fear of movement-related pain [18,32].

According to the fear-avoidance model, when a patient develops fear of movement-related pain, it is common to adopt avoidance behaviors. Although initially protective, this response may contribute to the maintenance of pain and the worsening of functional disability over time [13,14]. These results indicate that therapeutic strategies aimed at modulating the perceived threat associated with movement, such as graded motor imagery, gradual exposure, or in vivo exposure, may be useful in the rehabilitation of this population [80–82].

Future studies should investigate the impact of explicit motor imagery instructions during the viewing of movement pictures, as well as include self-report measures of fear of movement-related pain, perceived threat, and emotional reactivity [72,73]. It would also be valuable to separate neutral and negative shoulder pictures into distinct presentation blocks, enabling the examination of emotion-specific autonomic changes. Furthermore, longitudinal studies including people with varying degrees of chronicity and functional impairment may help elucidate how autonomic dysfunction interacts with central sensitization mechanisms and contributes to the persistence of shoulder pain.

## 5. Conclusion

Passively viewing shoulder movement pictures was associated with reduced parasympathetic activity and increased pain sensitivity in people with CSP. Our findings suggest that exposure to emotional stimuli may be associated with changes in autonomic responses and pain sensitivity, highlighting the potential involvement of the autonomic nervous system in these processes.

### Use of generative AI

Generative artificial intelligence (ChatGPT; OpenAI) was used to assist with language editing and clarity of expression during the manuscript revision process. The authors take full responsibility for the content of the manuscript, including the accuracy of all information, interpretations, and citations.

## Supporting information

S1 Table Description, mean and SD of valence and arousal of shoulder movement pictures representing daily activities (*N* = 24). (DOCX)

S1 Fig Spearman correlation matrix illustrating the associations between changes (during pictures minus before pictures) in autonomic modulation (HRV indices and baroreflex sensitivity), pressure pain threshold (PPT), and pain intensity. Correlation coefficients are presented within each cell. Blue indicates positive correlations, red indicates negative correlations, and darker colors represent stronger correlations.
(TIFF)

S2 File Inclusivity in global research questionnaire. (DOCX)
