# Supplementary material for: Heart rate variability and pain sensitivity in people with chronic shoulder pain during passive viewing of shoulder movement pictures
Source: PLoS One. 2026 Sep 28;21(9):e0350902. doi: 10.1371/journal.pone.0350902 (PMC13618931; doi:10.1371/journal.pone.0350902)
Supplement: Supplementary file 1 [file pone.0350902.s001.docx]

**Table S1.** Description, mean and SD of valence and arousal of shoulder movement pictures representing daily activities (*N*=24).

| **ICF code** | **Description** | **Valence Mean (SD)** | **Arousal Mean (SD)** |
| --- | --- | --- | --- |
| d4451 | Pushing | 2.19(2.18) | 7.33(2.42) |
| d4304 | Carrying on the head | 2.61(2.41) | 6.57(2.61) |
| d6403 | Using household appliances | 3.61(2.99) | 5.07(3.20) |
| d4303 | Carrying on shoulders | 2.66(2.39) | 6.92(2.6) |
| d4702 | Using public motorized transportation | 2.66(2.56) | 6.40(2.93) |
| d4200 | Transferring oneself while sitting | 2.8(2.22) | 6.11(2.74) |
| d4300 | Lifting | 2.92(2.09) | 6.23(2.66) |
| d6400 | Washing and drying clothes and garments | 2.97(2.13) | 6.07(2.93) |
| d5204 | Caring for toenails | 3.66(2.39) | 5.5(2.84) |
| d5401 | Taking off clothes | 3.73(2.37) | 6.09(2.73) |
| d6402 | Cleaning living area | 3.73(2.69) | 5.42(2.93) |
| d5400 | Putting on clothes | 3.88(2.45) | 5.8(2.54) |
| d4452 | Reaching | 4.26(2.82) | 4.9(3.15) |
| d5202 | Caring for hear | 4.26(3.05) | 4.9(2.92) |
| d6405 | Disposing of garbage | 4.40(2.53) | 4.61(2.64) |
| d4453 | Turning or twisting the hands or arms | 4.45(2.4) | 4.64(2.74) |
| d850 | Remunerative employment | 4.47(2.61) | 5.19(2.78) |
| d4301 | Carrying in the hands | 4.57(2.4) | 5.11(2.75) |
| d6404 | Storing daily necessities | 4.66(2.5) | 5(2.64) |
| d5102 | Drying oneself | 4.97(2.9) | 4.85(3.05) |
| d5101 | Washing whole body | 5.11(3.24) | 4.38(3.07) |
| d4450 | Pulling | 5.54(2.21) | 3.9(2.23) |
| d9203 | Crafts | 5.64(2.63) | 4.5(2.63) |
| d9200 | Play | 5.76(2.7) | 4.38(2.72) |

*Note - N (number of pictures); SD (standard deviation); ICF (International Classification of Functioning, Disability and Health)*

**Figure S1.** Spearman correlation matrix illustrating the associations between changes (Δ; during picture exposure minus before picture exposure) in autonomic modulation variables (HRV indices and BEI), PPT, and pain intensity. Numbers within each cell represent Spearman correlation coefficients (ρ). Blue indicates positive correlations, whereas red indicates negative correlations; darker shades represent stronger correlations and lighter shades weaker correlations. The autonomic modulation variables were generally correlated with one another, as were the PPT measures and the pain intensity measures. However, no associations between autonomic modulation and pain-related outcomes were found.
